# Space–Time Analysis of Peste des Petits Ruminants in Mali and Identification of Risk Factors

**DOI:** 10.1155/tbed/9903861

**Published:** 2024-12-19

**Authors:** Olivier Mahuton Zannou, Ahmadou Nouh Sow, Boundiala Sissoko, Cheick Oumar Fomba, Theodore J. D. Knight-Jones, Michel Dione

**Affiliations:** ^1^Animal and Human Health, International Livestock Research Institute, Nairobi, Kenya; ^2^National Directorate of Veterinary Services, National Directorate of Veterinary Services, Bamako, Mali; ^3^Animal and Human Health, International Livestock Research Institute, Addis Ababa, Ethiopia

**Keywords:** mali, PPR, risk factors, small ruminant, spatial and temporal

## Abstract

Livestock farming is an important part of Mali's economy and a major source of income for the rural population especially women. One of the major constraints to this activity is high burden of animal disease, in particular peste des petits ruminants (PPR), which hinder the productivity of small ruminants and thus reduces the income of livestock farmers. This disease that has an effective vaccine is subjected to a worldwide eradication program. The aim of this study is therefore to develop risk maps and identify the disease's risk factors to inform national vaccination strategy in Mali. This tool will help decisions-makers rationalize the limited resources available for disease control. A compilation of retrospective cases of PPR from 2011 to 2023 was used to generate risk maps using multivariable regression models and geographically weighted regression. Results show that the southern regions of Mali are more at risk than the northern. PRR cases occur more during rainy and hot dry seasons. Parameters such as railroads length, rainfall, and watering points were identified as risk factors for the spread of the disease. These results point out high priority areas during a risk-based vaccination campaign against PPR in Mali.

## 1. Introduction

Mali has a strong agropastoral tradition, and animal husbandry is an important activity in the family farm economy. Livestock is the main source of subsistence income for more than 30% of the population [1]. In total, 85% of rural households own ruminants, which reflects the economic and social importance of livestock in Mali [2]. Livestock production contributes 80% of the income of rural populations living in pastoral systems and 18% in agropastoral systems [2]. Livestock contributes 13.6% to gross domestic product (GDP), 24% to production in the rural sector, and nearly 20% to export earnings [1]. Mali's national herd is the largest in the West African Economic and Monetary Union (WAEMU) and the second largest in the Economic Community of West African States (ECOWAS) after Nigeria [3]. The small ruminant population of Mali in year 2020 was 20,142,677 heads of sheep and 27,810,553 heads of goats [3].

Despite its huge potential to create wealth hence reduce poverty, especially for rural communities, the Malian livestock sector faces major constraints. Disease outbreaks including Trypanosomiasis, peste des petits ruminants (PPR), Pasteurellosis, respiratory infections, brucellosis, etc. were the main constraints reported by farmers during a participatory survey carried out during a study investigating reproductive failures in small ruminants in Mali [4]. PPR was the second most frequently cited small ruminant disease by farmers during this participatory survey [4].

PPR is a highly contagious viral disease affecting small ruminants. This disease has now become endemic in Africa with multiple lineages [5], with localized and significant epidemic outbreaks, causing losses especially in young animals [6]. Each year, the economic losses caused by PPR are estimated at between 1.2 and 1.7 billion dollars and are mainly due to animal deaths, reduced production, and the cost of combating the disease [7]. An impact assessment of PPR in Senegal showed an annual reduction in farm profitability of 51%–61% with a hypothetical 25% incidence rate of PPR, corresponding to an average of $1051 to $1246 USD lost per household per year [8, 9]. A cost–benefit analysis study showed that eradicating PPR would be highly cost effective, yielding a net benefit of 74.2 billion dollars over 15 years [10]. This disease has been targeted by the global community for eradication by the end of 2030 [7]. PPR is a major concern for the livestock sector in Mali. Its occurrence can have severe consequences on the large part of the population who depend on livestock as a major source of income, especially women and small-scale farmers in rural areas [11].

Household income losses ranged from 02% to 40% of total annual income; percentages varied depending on the income scenario and on the gross annual economic impact of PPR on small ruminant production, which ranged from 20% to 80%, based on results of the bioeconomic model [12].

PPR has a very effective vaccine that allows it to be controlled and is even the basis of the eradication program initiated by the World Organisation for Animal Health (WOAH) and Food and Agriculture Organization (FAO) of the United Nations [7]. In Mali, the vaccine is produced by the Central Veterinary Laboratory (LCV) in Bamako for several years now and made available to private veterinarians for use upon request by small ruminant farmers and during mass ruminant vaccination campaigns organized by the government through the Directorate of Veterinary Services [13, 14].

Despite of many efforts by the government to vaccination small ruminants, the coverage rate is still very low (14.73%) according to the National Directorate of Veterinary Services (DNSV) [15]. This is due to the limited participation of farmers in vaccination campaigns. Many reasons have been put forward including the high cost of vaccination, the frequent vaccine shortages during the vaccination campaigns, women facing time constraints, and limited access to information about vaccination schedules and limited financial resources made available to the veterinary service [16]. In the face of this situation, there is need for a tool or a strategy to guide decision making to rationalize resources and optimize vaccination.

Therefore, a good understanding of the distribution of PPR outbreaks over time and space and the identification of the risk factors could inform strategic risk-based vaccination. Studies in this area do not exist in Mali and are therefore urgently needed.

This study will support the control and eradication of PPR, with a view of achieving the objectives set by the institutions in charge of animal disease management.

## 2. Materials and Methods

### 2.1. Data

#### 2.1.1. PPR Case Data

The PPR case data used in this study are those from 2011 to 2023, published on the World Animal Health Information System (WAHIS) website of the WOAH and in annual reports of veterinary services of Mali (Table 1). The data published on the WAHIS website are those provided by the veterinary services of each country and validated in a bilateral participatory process between the two institutions (WOAH and Veterinary Services). In Mali, the data are supplied to WOAH by the DNSV. Information on the location of outbreaks (villages) was collected in the DNSV annual reports from 2011 to 2023. This information made it possible to generate the Global Positioning System (GPS) coordinates of these disease outbreaks using Geographic Information System (GIS) tools including Mapcarta (https://mapcarta.com) and OpenStreetMap (https://www.openstreetmap.org). The village names were entered one by one in the search box of the above-mentioned websites, and the coordinates of these villages were returned after running.

#### 2.1.2. Environmental, Land Use, Livestock, and Climatic Data

In order to identify the risk factors associated with the occurrence of PPR outbreaks, we downloaded data such as road and rail networks, water courses and bodies, ground cover, elevation mask, climatic data such as rainfall (average monthly rainfall for 1970–2000), temperature, solar radiation, wind speed, etc. (Table 1).

### 2.2. Variable Preprocessing

We used the collinearity test on the predictors (variables) to check for linear associations between them. Two variables are perfectly collinear if there is an exact linear relationship between them. We used the variance inflation factor (VIF) for the diagnosis of collinearity of predictors of the incidence of PPR cases. This method allows us to see which variables are really necessary for the computation of the different models [18]. A VIF below 5 suggests a low collinearity. When the VIF is between 5 and 10, the collinearity is moderate and can be problematic. If the VIF rises above 10, it can be argued that the regression coefficients are mis-estimated because of multicollinearity, which must be treated appropriately [18, 19]. We removed step by step the variables with a VIF above 10 and check again to see if there is other with the same issues and if it can decrease the VIF of other remaining variables. Variables with VIF > 5 will not be used in this study. This helped in the selection of relevant predictors for the modeling (Table 1). Variables with many missing values were removed from the list of predictors. We the package “faraway” and the function “vif” in R.

### 2.3. Analysis

We used these variables to build a database. The R Statistical Software [20] was used for the descriptive analysis of the PPR database built. Various functions were used for these descriptive analyses namely “aggregate” and “summary” in packages “stats” and “base,” respectively. These functions helped to aggregate and summarize the data by month, season, and year. Incidence was calculated based on the annual population of small ruminants in each cercle. The mean and standard deviation of incidence are calculated by month, year, and season.

#### 2.3.1. Spatial Analysis

The cluster analysis was carried out in two steps. All the spatial analyses were performed with the outcome of cumulative incidence of PPR per 10,000 small ruminants per cercle.

We started by a global cluster analysis method (Moran's I) to check if there are some clusters in our study area [21]. The first step in a Moran's I analysis requires that we define “neighboring” polygons. Here, we will adopt a contiguous neighbor definition, and we choose the cercles of Mali as our neighboring polygons. The Mali shapefile we used contain 50 contiguous cercles so our polygons share at least one vertex; this is the “queen” case. Then we assigned weights to the neighbors. Our polygon here was assigned equal weight when computing the neighboring mean. The hypothesis we are testing states that “the PPR incidence are randomly distributed across cercles following a completely random process.” We used the Monte Carlo method to test this hypothesis with number of simulation of 999.

The second step was the determination of the locations of clusters with three different methods including local indicators of spatial association (LISA), Getis Ord, and Satscan.

The first method, the LISA, was used to detect outliers [22]. The LISA is implemented in the form of the Local Moran statistic to discover hot spots and cold spots (local clusters) in the data, as well as local spatial outliers. The main objective of this technic is to Identify clusters with the Local Moran cluster map and significance map and interpret the spatial footprint of spatial clusters. The function used in GeoDa software is “Univariate Local Moran's I”. To carry out the spatial autocorrelation analysis, we will need a spatial weights file. We loaded from a previous Moran's I analysis, the “Queen contiguity”. For this univariate analysis, we used the variable “PPR incidence”. We used a randomization option of 999 permutation and a confidence interval of 95% (*p*  < 0.05).

The second method, “Getis Ord” was used to identify locations surrounded by cluster of high or low values [23]. The Getis Ord statistic is derived from a point pattern analysis logic that consisted of a ratio of the number of observations within a given range of a point to the total count of points. In a more general form, the statistic is applied to the values at neighboring locations (as defined by the spatial weights). There are two versions of the statistic. They differ in that one takes the value at the given location into account, and the other does not [23]. In the software GeoDa, the following menu allows to implement the Getis Ord analysis: Space > Local G or Space > Local G^*⁣*^*∗*^^. The parameters used here are identical to the one used in the previous local statistics analysis (LISA).

The third method was “Satscan,” which can highlight spatial and temporal small and compact clusters [24]. We performed a space–time analysis using SaTScan software to detect PPR space–time clusters and to see if they are statistically significant. This can allow to test whether this disease is randomly distributed over space and time and evaluate the statistical significance of disease cluster alarms. For this purpose, we used three input files including “PPR Case file,” “PPR cases location file,” and “Small ruminant population file.” Other inputs data were the study period (2011 to 2023). For the analysis, the following options were selected: the type of analysis was space–time analysis, the probability model was “Space–Time permutation”; the time aggregation was “Month”; the maximum spatial cluster size was “50% of population at risk”; and the spatial window shape was “Cicular.” The output file selected was “Shapefile for GIS software” for mapping and “Text file” for details on the clusters.

The Moran's I, LISA, and Getis Ord methods were performed in R and GeoDa, and the SatScan analysis was performed with SaTScan v10.1.3 64-bit and QGIS 3.26.3.

We used the Grid (Nearest Neighbor) of GDAL in QGIS to draw risk maps of PPR cases in Mali.

#### 2.3.2. PPR Risk Factors Identification

We used the geographically weighted regression (GWR) to screen the spatial risk factors of PPR incidence. We used the shapefile of the administrative map of Mali at cercle level. This shapefile contains 50 administrative divisions (cercles). The dependent variable was the log-transformed PPR cumulative incidence at the cercle level and the predictors are climate, land use, livestock, geographical and environmental variables (Table 1). The GWR was performed in the free spatial analysis software GeoDa 1.8.16 [25] using step by step two models. First, we used “The classic ordinary least squares (OLS) regression” that allowed us to identify the presence of spatial dependence. The Jarque–Bera test in the OLS model helps to know if there was normality in errors and Moran's I and five other test help to know if there was spatial dependence. These statistics are the simple LM test for a missing spatially lagged dependent variable (Lagrange Multiplier [lag]), the simple LM test for error dependence (Lagrange Multiplier [error]), variants of these robust to the presence of the other (Robust LM [lag] and Robust LM [error]), which tests for error dependence in the possible presence of a missing lagged dependent variable, Robust LM (lag) is the other way round, and a portmanteau test (SARMA, in fact Lagrange Multiplier [error] + Robust LM [lag]). After identifying the presence of spatial dependence with this first model and according to the results of the various spatial dependence tests, we introduced spatial lag with the “Spatial Lag Model” or check with the “Spatial Error Model” to improve the model [26]. The variables included in the multivariable GWR model are the one that VIF are below 5 as described above. The best set of risk factors was identified with a multivariable model (*p*  < 0.05). We also checked the multicollinearity of the GWR model and removed accordingly variables to have a score below 20. The software that used these various variables predict the log-transformed PPR cumulative incidence. The predicted values of the log-transformed incidence and the errors were inverse log-transformed and used to generate maps in QGIS.

We used a logistic Poisson regression to search for the seasonality of PPR outbreaks. For that purpose, we created a variable “Season” based on the date of occurrence of the outbreaks of PPR. We use the function “glm” for a logistic regression with family “Poisson”of the package “tidyverse” to compare the occurrence of PPR cases throughout the various seasons of Mali.

## 3. Results

### 3.1. Descriptive Statistics

Five thousand (5000) PPR cases have been reported from 2011 to 2023 in Mali from 07 regions and 15 cercles (Table 2). The minimum cases per outbreak were 03, and the maximum were 920. The median (interquartile range [IQR]) value of PPR cases was 41 (20.25–177.50). The median PPR cumulative incidence (per 10,000 small ruminants) was 1.15 (0.36–4.65).

The highest average incidence of PPR cases for 10,000 small ruminants was reported in 2018 (13.80), during July (13.83), during the hot dry season (5.89), and in the region of Koulikoro (9.82).

### 3.2. Clustering and Risk Prediction

**Figure 1:**
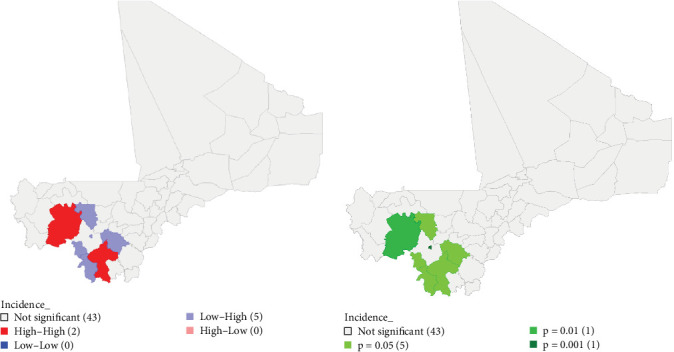
(A) Peste des petits ruminants clusters and outliers map based on overall cumulative incidence. High–High (in red) represent clusters with high numbers of cases in cercles with high numbers of cases in surrounding cercles. Low–Low (in blue) represent clusters with low numbers of cases in cercles with low numbers of cases in surrounding cercles. High–Low (in light red) represent outliers with high numbers of cases in cercles with low numbers of cases in surrounding cercles. Low–High (in light blue) represent outliers with low numbers of cases in cercles with high numbers of cases in surrounding cercles. The gray color represents areas without significant cluster or outlier. Number in parenthesis represents the number of cercle belonging to this type of clusters or outliers. (B) Peste des petits ruminants clusters and outliers significance map based on overall cumulative incidence. The various green colors represent the *p*-values of significant clusters and outliers. The gray color represents areas without significant cluster or outlier. Number in parenthesis represent the number of cercle with the same *p*-value.

The global cluster analysis with Moran's I (I: −0.012, *Z*-value: 01691; *p*-value: 0.276) did not reveal a global cluster of PPR cases in Mali. The local cluster analysis with LISA revealed the presence of two (02) significant high–high (high numbers of cases in cercles with high numbers of cases in surrounding cercles) clusters in the cercles of Bougouni (*p*=0.05) in the region of Sikasso and Kita (*p*=0.001) in the region of Kayes (Figure 1A, B).

It also revealed five (05) low–high (low numbers of cases in cercles with high numbers of cases in surrounding cercles) outliers, located in the cercles of Bamako (*p*=0.001), Dioïla (*p*=0.05), Kolokani (*p*=0.05), Kangaba (*p*=0.05), and Yanfolila (*p*=0.05).

**Figure 2:**
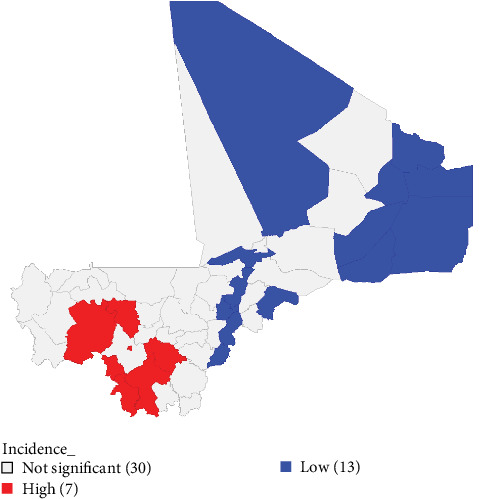
Peste des petits ruminants hotspots map based on overall cumulative incidence. Locations in red represent hotspots that are cercles with high risk of occurrence of PPR cases. Locations in blue represent coldspots that are cercles with low risk of occurrence of PPR cases. The gray colour represents areas without significant spots. Number in parenthesis represents the number of cercle belonging to this group.

The analysis of locations with high risk (hot spots) and low risk (cold spots) with Getis Ord methods revealed, with confidence interval of 95% (*p*  < 0.05), that there was a high risk of PPR cases in seven (07) cercles and low risk in thirteen (13) cercles (Figure 2). The hot spots are located in Bamako (*p*=0.05), Bougouni (*p*=0.05), Dioïla (*p*=0.05), Kangaba (*p*=0.05), Kita (*p*=0.01), Kolokani (*p*=0.05), and Yanfolila (*p*=0.05). The cold spots are located in Abeïbara (*p*=0.001), Ansongo (*p*=0.001), Djenné (*p*=0.001), Gao (*p*=0.001), Kidal (*p*=0.001), Ménaka (*p*=0.001), Mopti (*p*=0.001), Niafunké (*p*=0.001), Tominian (*p*=0.001), Koro (*p*=0.001), Tin-Essako (*p*=0.001), Tombouctou (*p*=0.001), and Yorosso (*p*=0.001).

**Figure 3:**
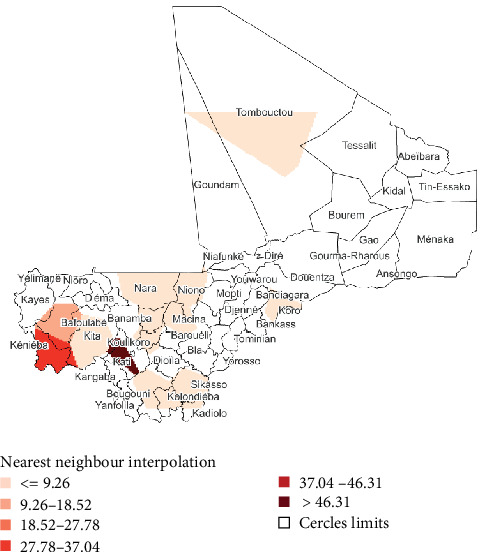
Peste des petits ruminants risk maps based on overall cumulative incidence.

The risk map showed that the cercles of Kati and Kéniéba are at the highest risk. The highest risk areas are in the south of the country, while the north-east side is at low risk of PPR cases (Figure 3).

**Figure 4:**
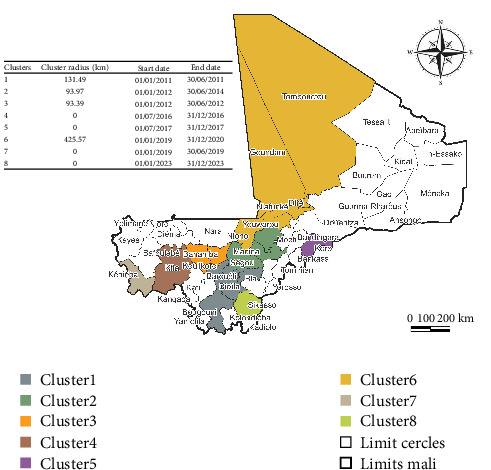
Significant space–time clusters of peste des petits ruminants cases reported in Mali from January 2011 to December 2023.

The retrospective space–time analysis scanning for high-rated clusters using the space–time permutation model in SatScan software revealed eight (08) clusters. These eight (08) clusters included twenty-one (21) cercles. Two (02) clusters were detected in 2012 and 2019 and one (01) in 2011, 2016, 2017, and 2023. The largest clusters were detected in 2019 with a radius of 425.57 km and included six (06) cercles. The smallest clusters contain only one cercle. The longest lasting cluster lasted thirty (30) months. The shortest-lasting clusters lasted six (06) months (Figure 4 and Table S1). Table S1 in Supporting information summarizes all information about each cluster including the cercles belonging to each cluster, the radius of the cluster, the start and end dates of the cluster, and the ratio observed/expected cases of PPR in the cluster and the test statistics.

### 3.3. Risk Factors Associated With PPR Cases

With the OLS model, a stepwise method helped to select predictors to keep in the GWR model to have a multicollinearity score below 20. The selected predictors included railroad length, rainfall, land cover, elevation, water areas, water lines, and sheep and goats' density.

The ordinary least squares (OLS) regression indicated non-normality of the error because the Jarque–Bera test result was significant. The Moran's I score of −2.29 is highly significant (*p*  < 0.05), indicating strong spatial autocorrelation of the residuals. The LM lag, the LM error, and the LM SARMA are also significant indicating the presence of spatial dependance of PPR cumulative incidences. The significance of the LM Lag, the LM error, and the robust LM-error suggested running the “spatial error model” to improve the model.

The spatial error model in the multivariable geographically weighted regression revealed that railroads length, rainfall, and water areas are associated to the incidence of PPR cases in Mali (Table 3).

**Figure 5:**
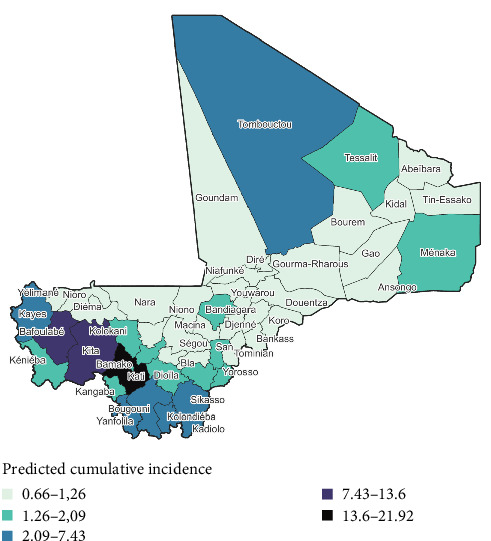
Risk map based on overall predicted cumulative incidence of PPR from geographically weighted regression model.

**Figure 6:**
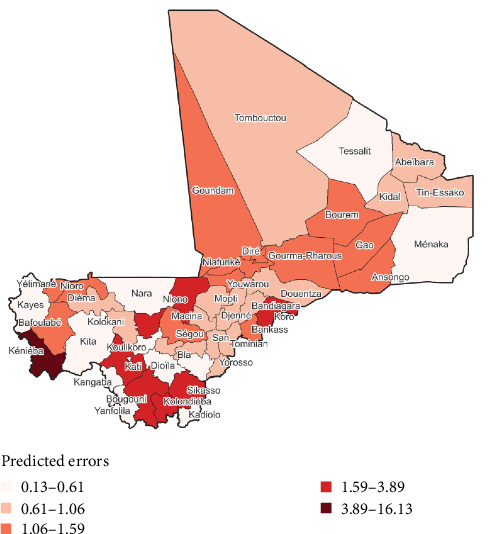
Mali's map showing the overall cumulative incidence of PPR prediction errors from geographically weighted regression model.

The two highest predicted PPR cumulative incidence per cercles were observed, respectively, in Kati (21.92 per 10.000 head of small ruminants) and Kita (13.60 per 10.000 head of small ruminants). The two lowest predicted PPR cumulative incidence per cercles were observed, respectively, in Goundam (0.660 per 10.000 head of small ruminants) and Yélimané (0.668 per 10.000 head of small ruminants) (Figures 5 and 6).

The logistic regression model revealed that the hot dry season and the rainy season are significantly associated with PPR outbreaks in Mali (Table 4).

## 4. Discussion

This study has highlighted the hot spots and concentration points of PPR outbreaks in time and space. This information will be very useful in achieving the objectives of PPR eradication, which recommends risk-based vaccination [7]. PPR is one of the most important diseases for small ruminant farmers [8, 9, 27]. This disease has an effective vaccine for its control [28]. The availability of additional information, particularly on areas with high concentrations of PPR cases, can help the authorities refine other vaccination strategies and make better use of the limited resources allocated to animal disease control.

Spatial analysis shows a high concentration of PPR outbreaks in the southern part of the country. The cercles of Bamako, Bougouni, Dioïla, Kangaba, Kita, Kolokani, and Yanfolila represent clusters of concentrated PPR cases and hot spots for the occurrence of this disease over the entire study period in Mali. These cercles are located in the wettest areas of Mali, and the density of small ruminants in these areas is among the highest in the country. The wettest areas were detected by the present study as factors favoring the occurrence of the disease. These are also areas of intense commercial transactions between Mali and its southern neighboring countries. Mali has one of the largest livestock populations in West Africa. It is the second largest livestock-producing country in the economic community of West African States zone, after Nigeria, and the first in the WAEMU zone [3]. Mali's livestock is exported live to neighboring countries such as Senegal, Côte d'Ivoire, Togo, Benin, Ghana, and Guinea [29].

Spatial factors such as railroads, rainfall, and the presence of water areas have been identified as risk factors for the occurrence of the disease.

The transportation of livestock by railroads could be a factor in the spread of the disease if biosecurity measures are not taken when moving livestock between rail-served areas. Bonniewell has described in one of his studies that mass transportation of small ruminants from various origins is a risk factor for the contamination and spread of the PPR virus [30].

Small ruminant farming is mostly practiced in an agro-pastoralist or pastoralist system in West Africa. This type of farming requires a constant search for natural forage and water. Areas with good rainfall are more favorable for this type of farming, but also for pathogens multiplication. During rainy seasons, small ruminants are confined to avoid crops destruction. With the arrival of the cool or rainy seasons, the temperature and humidity are favorable to the virus and increase its survival time [31]. The confinement allows close contact of many animals and favor pathogens transmission from one to another. Animals that have just survived a long period of drought are often thin and weak. Their weakened immune defenses render them susceptible to pathogens and benefit the virus. Epizootic peaks are frequent and numerous. These areas are also better supplied with natural fodder and periodically attract extensive or transhumant herds. This favors the mixing of several herds of different health status and the transmission of contagious animal diseases.

PPR is mainly transmitted through close contact, when a susceptible animal inhales the virus from the coughs and sneezes of infected animals. Transmission can also occur indirectly through contact with infected objects such as feed troughs, feed, pasturage, and bedding [32]. Sources of PPR virus are secretions from the eyes, nose, and mouth of infected animals, as well as their feces [33, 34]. High density of PPR virus host can favor close contact and then increase the transmission and spread of the virus. Perennial watering points are sources of water for ruminant herds. They are permanent meeting points for animals, and in areas of high animal density, they are sources of contagious diseases such as PPR. Nkamwesiga et al. [35] in 2023 found the same risk factor in their study when they compared the prevalence of PPR cases between villages where animals are watered at swamps and villages without swamps. Swamps are watering points used by all animals in a village to drink water and this consequently increases the chances of interacting with PPRV infectious flocks [35]. Other studies have also confirmed that collective watering point are risk factors for contamination and spread of infectious and transmissible diseases [36, 37].

The hot dry season and the rainy season have been identified as periods associated with PPR epidemics. During the hot dry season, animals are subjected to high levels of stress associated with poor feeding, which could favor the development of infections more rapidly. During the rainy season, animals are confined to avoid the destruction of crops planted by farmers. During this period, hygiene conditions are poorly respected, favoring the development of contagious infectious diseases such as PPR. Obi et al. [38] cited by Mantip, Shamaki, and Farougou [30], showed in a study carried out in western Nigeria that PPR cases may occur almost throughout the year, with peaks in the wet months. Niu et al. [39] also found the same relationship between precipitation and PPR outbreaks in their study on modeling the distribution of PPR outbreaks with meteorological data. A study in India, on the other hand, shows that the disease occurs in all seasons, but with greater frequency during the lean season [40].

PPR has effective vaccines that are used in disease control and eradication plans.

Given the significant negative impact that PPR can have on the food security and livelihoods of poor farmers, who are the main keepers of sheep and goats, the Global Steering Committee of the Global Framework for the Progressive Control of Transboundary Animal Diseases (GF-TADs) in 2012, the FAO of the United Nations, and the World Organization for Animal Health (OIE) jointly initiated the development of a global strategy for the control and eradication of PPR. This initiative resulted in a Global Strategy for the Eradication of PPR by 2030, which was presented at the FAO and OIE International Conference for the Control and Eradication of Peste des petits ruminants held in Côte d'Ivoire, in April 2015 [7]. This strategy recommends during its implementation periodic and constant assessments of the epidemiological situation of the disease. The results of these assessments will help to carry out targeted vaccination campaigns to efficiently manage the related resources.

The results of this study will be used to reinforce the measures needed to improve vaccination coverage in the areas covered by the highlighted clusters in Mali. It is a decision-support tool for Mali's veterinary services, enabling them to effectively direct resources toward PPR control.

The recent development of a thermotolerant vaccine by the Laboratoire Centrale Vétérinaire de Bamako, with technical and financial support from ILRI, will make a substantial contribution to this effort [41]. This new vaccine removes the constraint of the cold chain and facilitates the vaccination of herds far from vaccine storage facilities. Better organization of small ruminant value chain stakeholders in participation in vaccination through innovation platforms would also be a tool for improving PPR vaccination coverage [16].

## 5. Conclusion

This study gives a better idea of the distribution in time and space of PPR outbreaks and their risk factors. It is an excellent decision-making tool for animal disease management authorities, enabling them to make optimum use of the limited resources allocated to animal epidemic control. When the nature and distribution of the risk factors for transmission and persistence of a pathogen are known, it becomes possible to pinpoint adapted surveillance and control measures to high-risk contexts, maximizing impact and minimizing costs. The results of this study will enable better orientation of strategies to achieve the goal of PPR eradication.

## Figures and Tables

**Table 1:** Type of variables used in the study and their sources.

Variables	Variable categories	VIF	Data sources	References and links
PPR cases	Biological data	—	WAHIS and DNSV-Mali	https://wahis.woah.org DNSV-Mali
Rainfall	Climatic	4.27	WorldClim	https://www.worldclim.org
Temperature	Climatic	2.91	WorldClim	https://www.worldclim.org
Road length	Land use	2.14	Diva-Gis	https://diva-gis.org
Railroad length	Land use	1.47	Diva-Gis	https://diva-gis.org
Water bodies	Environmental	1.89	Diva-Gis	https://diva-gis.org
Water lines	Environmental	4.29	Diva-Gis	https://diva-gis.org
Land cover	Environmental	3.22	Diva-Gis	https://diva-gis.org
Elevation	Environmental	1.59	Diva-Gis	https://diva-gis.org
Sheep and Goat density	Livestock	1.50	Gridded Livestock of the Wworld—2015 (GLW 4)	https://www.fao.org/livestock-systems/en/ https://dataverse.harvard.edu/dataverse/glw_4 [17]

**Table 2:** Peste des petits ruminants cases based on passive surveillance data reported in Mali from 2011 to 2023.

Variables	PPR cases	Average incidence (10,000 SR^a^)	Standard deviation
Year			
2011	393	3.08	2.47
2012	393	3.91	3.66
2013	45	0.38	0.28
2014	34	0.15	0.03
2016	106	5.33	NA^b^
2017	417	1.07	0.78
2018	1869	13.80	11.11
2019	610	5.59	8.39
2020	32	0.20	NA
2021	35	0.36	0.22
2022	803	6.21	8.73
2023	263	1.51	0.64
Month			
April	290	2.93	2.49
August	188	0.71	0.07
December	448	1.98	2.44
February	1183	3.47	4.82
January	167	0.91	1.03
July	935	13.83	19.13
June	225	3.91	2.57
March	1364	7.91	8.21
May	180	2.60	NA
November	20	0.51	NA
Season			
Cool dry season	1818	2.27	3.48
Hot dry season	1834	5.89	6.76
Rainy humid season	1348	4.79	9.29
Region			
Bamako	18	0.15	NA
Kayes	1197	7.61	6.37
Koulikoro	2004	9.82	10.59
Mopti	295	2.24	NA
Ségou	461	0.52	0.58
Sikasso	845	1.70	2.51
Tombouctou	180	2.60	NA
Total	5000	—	—

^a^Small ruminant.

^b^Not applicable (single incidence value).

**Table 3:** Variables associated with a log-transformed overall cumulative incidence of peste des petits ruminants in Mali using multivariable geographically weighted regression analysis.

Variables	Coefficient	Std. error	*p*-Value
Constant	−6.40e−1	1.72e−1	0.00018
Rainfall	5.53e−4	1.12e−4	0.00000
Landcover	4.96e−9	1.63e−7	0.97569
Elevation	8.44e−4	5.24e−4	0.10708
Small ruminant density	−9.76e−7	9.34e−6	0.91683
Water areas	2.45e−11	1.30e−11	0.05392
Water lines	7.90e−8	4.38e−8	0.07111
Length of railroads	1.60e−6	2.52e−7	0.00000

**Table 4:** Influence of seasons on PPR cases in Mali.

Variable	Coefficients	Odds ratios	Standard error	*z* value	Pr (>|z|)
(Intercept)	1.30	3.68	0.02	55.56	<2e−16*⁣*^*∗∗∗*^
Hot dry season	2.19	8.90	0.03	66.05	<2e−16*⁣*^*∗∗∗*^
Rainy season	1.88	6.54	0.04	52.25	<2e−16*⁣*^*∗∗∗*^

*Note:* Significance codes: 0 “*⁣*^*∗∗∗*^” 0.001 “*⁣*^*∗∗*^” 0.01 “*⁣*^*∗*^” 0.05 “.” 0.1 “ ” 1.

## Data Availability

The data that support the findings of this study are available from the corresponding author upon reasonable request.

## Supporting Information

Supporting Information Additional supporting information, on the significant space-time clusters of Peste des petits ruminants cases reported in Mali from January 2011 to December 2023, can be found online in the Supporting Information section.
