# Supplementary material for: Space–Time Analysis of Peste des Petits Ruminants in Mali and Identification of Risk Factors
Source: Transbound Emerg Dis. 2024 Dec 19;2024:9903861. doi: 10.1155/tbed/9903861 (PMC12016892; doi:10.1155/tbed/9903861)
Supplement: Supporting Information — Additional supporting information, on the significant space-time clusters of Peste des petits ruminants cases reported in Mali from January 2011 to December 2023, can be found online in the Supporting Information section. [file 9903861.f1.docx]

**Supplementary files**

S1. Significant space-time clusters of Peste des petits ruminants cases reported in Mali from January 2011 to December 2023.

| Cluster (Cercles) | Coordinates^[[1]](#footnote-1)^ | Radius^[[2]](#footnote-2)^ (Km) | Start date | End date | O/E^[[3]](#footnote-3)^ | LLR^[[4]](#footnote-4)^ | P-value |
| --- | --- | --- | --- | --- | --- | --- | --- |
| Diré, Goundam, Niafunké, Niono, Tombouctou, Youwarou | 18.606610 N, 4.991293 W | 425.57 | 2019/1/1 | 2020/12/31 | 7.79 | 518.66 | <0.001 |
| Koro | 14.299633 N, 2.813404 W | 0 | 2017/7/1 | 2017/12/31 | 11.99 | 469.90 | <0.001 |
| Barouéli, Bla, Bougouni, Dioïla, Koulikoro | 12.367756 N, 6.773613 W | 131.49 | 2011/1/1 | 2011/6/30 | 12.09 | 464.17 | <0.001 |
| Sikasso | 11.503860 N, 5.913418 W | 0 | 2023/1/1 | 2023/12/31 | 11.99 | 371.49 | <0.001 |
| Kita | 13.230625 N, 9.434046 W | 0 | 2016/7/1 | 2016/12/31 | 47.17 | 305.83 | <0.001 |
| Djenné, Macina, Ségou, Ténenkou | 13.990883 N, 5.373022 W | 93.97 | 2012/1/1 | 2014/6/30 | 8.86 | 234.27 | <0.001 |
| Banamba, kolokani | 13.781483 N, 8.160191 W | 93.39 | 2012/1/1 | 2012/6/30 | 12.72 | 151.58 | <0.001 |
| Kéniéba | 12.706650 N, 11.014226 W | 0 | 2019/1/1 | 2019/6/30 | 5.070 | 149.83 | <0.001 |

1. Coordinates of the centre of the cluster [↑](#footnote-ref-1)
2. Radius of the cluster [↑](#footnote-ref-2)
3. Observed cases divided by the expected cases in the cluster [↑](#footnote-ref-3)
4. Log likelihood ratio test statistics [↑](#footnote-ref-4)
